# Single-cell visualization of *mir-9a* and *Senseless* co-expression during *Drosophila melanogaster* embryonic and larval peripheral nervous system development

**DOI:** 10.1093/g3journal/jkaa010

**Published:** 2020-12-22

**Authors:** Lorenzo Gallicchio, Sam Griffiths-Jones, Matthew Ronshaugen

**Affiliations:** 1 School of Biological Sciences, Faculty of Biology, Medicine and Health, The University of Manchester M13 9PT, UK; 2 School of Medical Sciences, Faculty of Biology, Medicine and Health, The University of Manchester M13 9PT, UK

**Keywords:** microRNA, *mir-9a*, *Senseless*, peripheral nervous system, embryogenesis, wing disc

## Abstract

The *Drosophila melanogaster* peripheral nervous system (PNS) comprises the sensory organs that allow the fly to detect environmental factors such as temperature and pressure. PNS development is a highly specified process where each sensilla originates from a single sensory organ precursor (SOP) cell. One of the major genetic orchestrators of PNS development is *Senseless*, which encodes a zinc finger transcription factor (Sens). Sens is both necessary and sufficient for SOP differentiation. *Senseless* expression and SOP number are regulated by the microRNA *miR-9a*. However, the reciprocal dynamics of *Senseless* and *miR-9a* are still obscure. By coupling single-molecule FISH with immunofluorescence, we are able to visualize transcription of the *mir-9a* locus and expression of Sens simultaneously. During embryogenesis, we show that the expression of *mir-9a* in SOP cells is rapidly lost as *Senseless* expression increases. However, this mutually exclusive expression pattern is not observed in the third instar imaginal wing disc, where some *Senseless*-expressing cells show active sites of *mir-9a* transcription. These data challenge and extend previous models of *Senseless* regulation and show complex co-expression dynamics between *mir-9a* and *Senseless*. The differences in this dynamic relationship between embryonic and larval PNS development suggest a possible switch in *miR-9a* function. Our work brings single-cell resolution to the understanding of dynamic regulation of PNS development by *Senseless* and *miR-9a*.

## Introduction

One of the most impressive demonstrations of developmental robustness is the specification of the *Drosophila melanogaster* peripheral nervous system (PNS), which comprises all the organs that allow the fly to detect movement, pressure, temperature, and more. *Drosophila* sensilla number and position exhibit little or no variation from individual to individual, even in diverse environmental conditions (Hartenstein 1988). The key early developmental step involves the selection and specification of sensory organ precursor (SOP) cells from a field of equipotent cells. During early embryogenesis (∼5 h from fertilization), groups of epidermal cells start to express Achete–Scute complex genes. These proneural genes impart the potential to become neurons (Jarman *et al.* 1993; Jan and Jan 1994; Goulding *et al.* 2000; Huang *et al.* 2000; Reeves and Posakony 2005). This potential is then constrained via Notch lateral inhibition to a single cell in the cluster, the SOP cell (Ghysen and Dambly-Chaudiere 1989; Artavanis-Tsakonas and Simpson 1991). The many different classes of sensory organs all originate from SOPs and develop via common shared rounds of cellular division (Lai and Orgogozo 2004). The eventual differences between sensory organs arise in part through subsequent changes in cell death and proliferation (Orgogozo *et al.* 2001; Orgogozo and Schweisguth 2004).

One of the major effectors of PNS development is a gene named *Senseless* (*sens*) (Nolo *et al.* 2000). *sens* encodes a transcription factor (Sens) whose expression is initially activated and subsequently maintained by the proneural genes *achete* and *scute* (Jafar-Nejad *et al.* 2006). *sens* in turn maintains the expression of proneural genes to direct proper neuronal cell differentiation (Nolo *et al.* 2000; Acar *et al.* 2006). *sens* expression is first detectable during stage 10 of *Drosophila* embryogenesis, as isolated cells start to specify according to their SOP fate potential. As embryogenesis proceeds, these isolated *sens*-expressing SOPs ultimately give rise to the entire sensory organ. *sens* expression becomes repressed around stage 13, when SOPs are fully specified (Nolo *et al.* 2000). Since *sens* maintains proneural gene activation, loss-of-function *sens* mutant embryos exhibit a decreased number of SOPs, corresponding to a loss of sensory organs in the adult fly (Nolo *et al.* 2000). Gain of function mutations and ectopic expression of Sens cause an increased number of SOPs and consequently sensory organs (Jafar-Nejad *et al.* 2003; Li *et al.* 2006). Therefore, it is suggested that *sens* is necessary and sufficient for SOP differentiation (Nolo *et al.* 2000). The robustness and reproducibility of sensory organ development between individuals implicates *sens* as a keystone gene whose fine-scale regulation involves multiple feedback inputs.

Neurogenesis is extensively regulated by microRNAs (miRNAs) (Nolo *et al.* 2000; Hilgers *et al.* 2010; Caygill and Brand 2017). These small regulators of translation and mRNA stability contribute to the robustness of many biological processes. It has been shown that *miR-263a/b* stabilize sensory organ patterning in the retina by inhibiting sensory organ cell apoptosis (Hilgers *et al.* 2010), and that *miR-7* stabilizes neuronal differentiation in the *Drosophila* larval brain by targeting the Notch pathway (Caygill and Brand 2017). In addition, Li *et al.* (2006) showed that *miR-9a* regulates Sens function through multiple target recognition sites in the *sens* 3′ UTR. When *sens*′ *miR-9a* binding sites are mutated, Sens levels are not only higher but more sensitive to temperature perturbations (Cassidy *et al.* 2013), resulting in an altered distribution of sensory organs in the wing margin (Cassidy *et al.* 2013; Giri *et al.* 2020). Loss of function and overexpression of *miR-9a* produce opposite phenotypes with respect to *sens* in both embryos and larvae. Thus, the phenotypic consequences of *miR-9a* disruption mirror those of *sens*, suggesting that *miR-9a* is necessary to ensure appropriate Sens expression in the right cells and at the right level to convey robustness to SOP specification (Li *et al.* 2006).

The *miR-9a* miRNA is a member of one of the ∼30–40 families that are predicted to pre-date the divergence of protostomes and deuterostomes, and therefore to be conserved in essentially all bilaterian animals (Wheeler *et al.* 2009; Ninova *et al.* 2014). In every animal where *miR-9* family members have been studied functionally, they are found to regulate processes related to neurogenesis and neuronal progenitor proliferation (Sempere *et al.* 2004; Wheeler *et al.* 2009; Delaloy *et al.* 2010). For instance, overexpression of *miR-9* in zebrafish embryo (Leucht *et al.* 2008), mouse embryonic cortex (Zhao et al. 2009), and chicken spinal cord (Otaegi *et al.* 2011) all lead to a reduction of the number of proliferating neural progenitors and impairment of PNS development. These studies demonstrate clear similarities between *miR-9* expression and function in *Drosophila* and vertebrates. Disrupted *miR-9* function has also been linked with some human pathologies, including cancer progression (Nowek *et al.* 2018) and neurodegenerative amyloid diseases (Packer *et al.* 2008). For instance, tumorigenic cells from medulloblastoma appear to have decreased expression of *miR-9*, while a subclass of glioblastoma tumor cells express *miR-9* at a higher level (Ferretti *et al.* 2009; Kim *et al.* 2011). In addition, *miR-9* has been also found to have a role as a proto-oncogene and/or as a tumor-suppressor gene during progression of cancers not directly related with the nervous system (Coolen *et al.* 2013).

The current model of *miR-9a* function in *Drosophila* SOP specification suggests mutually exclusive reciprocal expression of *miR-9a* and *sens* in SOPs (Li *et al.* 2006). This in turn suggests a role for *miR-9a* in ensuring that only one of the cells in the progenitor field takes on SOP identity. In this work, we use single-cell quantitative fluorescent *in situ* hybridization (FISH) and nascent transcript FISH to investigate the *miR-9a*/Sens/SOP regulatory model in hitherto unseen detail. This use of single-molecule FISH (smFISH) coupled with immunofluorescence (IF) allows us to simultaneously visualize active sites of *miR-9a* transcription and Sens protein in both embryos and larval wing disc at the single-cell level. We use these data to analyze the dynamics of *miR-9a* transcription and Sens protein abundance. We find that *miR-9a* and Sens are initially co-expressed but ultimately exhibit a dynamic reciprocal expression pattern. We observe that *miR-9a* transcription becomes rapidly repressed in high Sens-expressing SOPs during embryogenesis, presumably as Sens protein accumulates in the cell nucleus. A subtly different co-expression dynamic was observed during wing disc development, where many SOPs also express *miR-9a*. These SOPs exhibit an inverse relationship between Sens abundance and *miR-9a* transcription. These new data refine and expand the previous model to provide key new insights into *miR-9a/sens* regulation in PNS development (Li *et al.* 2006). In particular, we include for the first time a temporal element to the understanding of the dynamics of *miR-9a* regulation of SOP differentiation.

## Methods

### Fly stocks, embryo collection, and fixing and larval dissection

Flies were grown at 25 or 18°C. Embryos were collected after ∼20 h and fixed in 1 V heptane + 1 V 4% formaldehyde for 30 min shaking at 220 rpm. The embryos were then washed and shaken vigorously for one minute in 100% methanol. Fixed embryos were stored in methanol at −20°C. Larvae were dissected in 1× PBS, carcasses were fixed in 1 V 1× PBS + 1 V 10% formaldehyde for ∼1 h, washed with methanol, and stored in methanol at −20°C.

Genotypes used for this study are: W [1118], Bloomington stock 3605 and 2XTY1-SGFP-V5-preTEV-BLRP-3XFLAG-Sens, VDRC stock ID 318017.

### Probe design, smFISH, and IF

We adapted the inexpensive version (Tsanov *et al.* 2016) of the conventional smFISH protocol in *Drosophila* (Trcek *et al.* 2017). Primary probes were designed against the mature *sens* and sfGFP mRNA and a genomic region flanking the *mir-9a* gene locus, all from FlyBase, using the Biosearch Technologies Stellaris probe Designer (version 4.2). To the 5′ end of each probe was added the Flap sequence CCTCCTAAGTTTCGAGCTGGACTCAGTG. Multiple secondary probes that are complementary to the Flap sequence were tagged with fluorophores (CAL Fluor Orange 560, CAL Fluor Red 610, Quasar 670) to allow multiplexing. Antibodies used were Anti Green Fluorescent Protein rabbit igG fraction (Invitrogen #A11122) at 1:500, anti-Sens (Nolo *et al.* 2000) at 1:1000, Goat anti-Guinea Pig IgG (H + L) Highly Cross-Adsorbed Secondary Antibody Alexa Fluor 555 (Invitrogen #A21435) at 1:500, and Goat anti-Rabbit IgG (H + L) Cross-Adsorbed Secondary Antibody Alexa Fluor 488 (Invitrogen #A11008) at 1:500.

### Imaging and quantification

Imaging was performed using a Leica SP8 Inverted Tandem Head confocal microscope with LAS X v.3.5.1.18803 software (University of Manchester Bioimaging facility), using 20×, 40×, and 100× magnifications. Deconvolution was performed using Huygens Pro v16.05 software. Protein fluorescence levels were measured using FIJI for Macintosh. From each picture, five measurements of background mean intensity were taken. Each single-cell measurement was then adjusted using the formula: integrated density of nucleus – (area of nucleus × background mean).

### Data availability

Strains and plasmids are available upon request. The authors affirm that all data necessary for confirming the conclusions of the article are present within the article, figures, and tables.

Supplementary material is available at G3.

## Results

### *miR-9a* is expressed in the dorsal ectoderm during embryogenesis and ubiquitously in the wing disc

In order to understand the interaction between *miR-9a* and its target *Senseless* (*sens*), we first systematically describe the *mir-9a* expression pattern in the embryo and imaginal disc at the single-cell level. Imaging mature miRNAs is difficult. Previously applied techniques require amplification and often have significant background noise problems (*e.g.* probes labeled with digoxigenin) (Biryukova *et al.* 2009). Many researchers have tried to overcome this (Lu and Tsourkas 2009), but these approaches still have very limited use for single-cell analysis and quantification.

**Figure 1:**
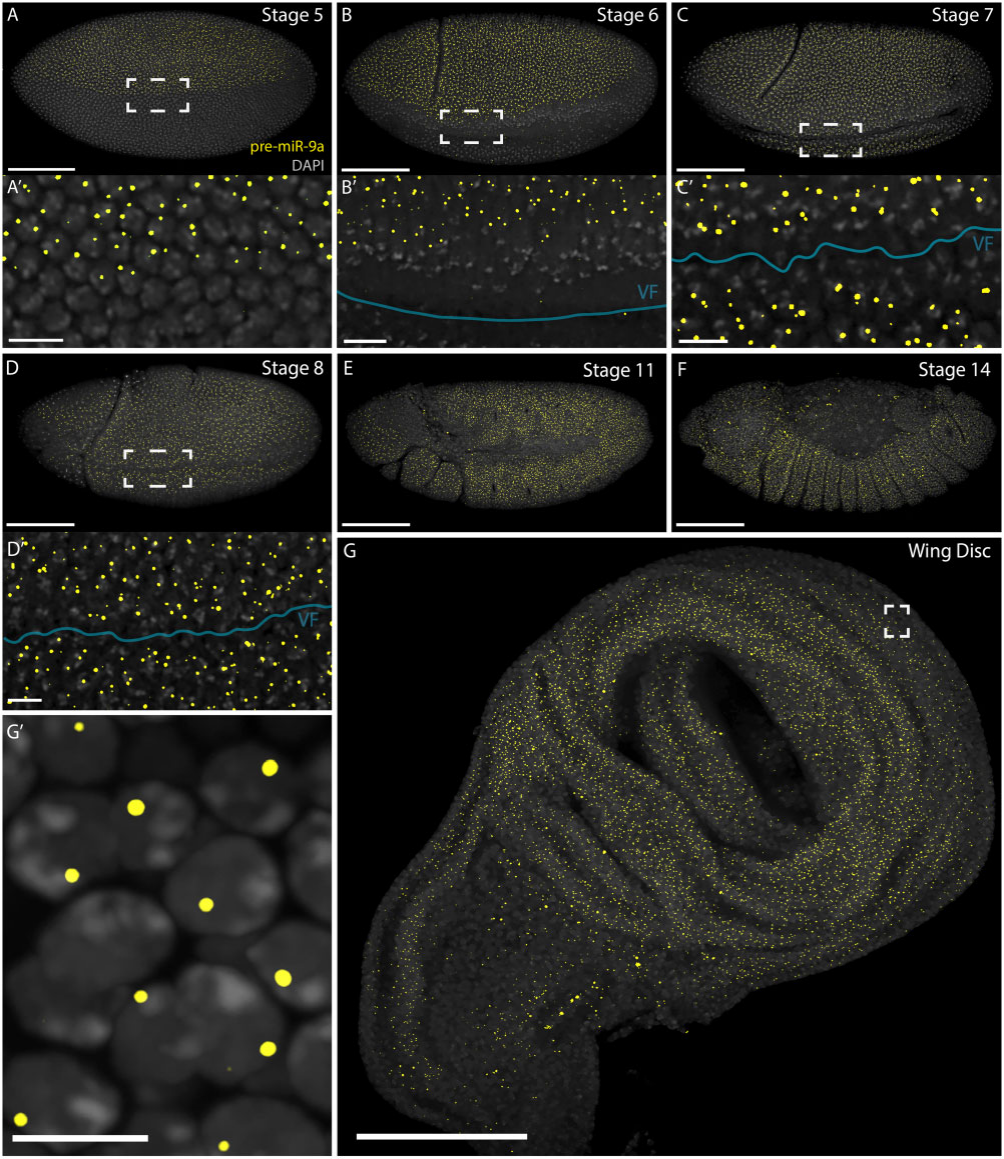
*mir-9a* expression pattern during embryogenesis and in the wing disc. Cells that are actively transcribing *mir-9a* present one or two puncta, indicating *mir-9a* active TSs. (A, A′) Stage 5 WT *Drosophila* embryo: *mir-9a* is expressed in the dorsal ectoderm, before the ventral furrow is evident. (B, B′) Stage 6 WT: the *mir-9a* expression domain extends toward the forming ventral furrow (highlighted with a blue line). (C, C′) Stage 7 WT. (D, D′) Stage 8 WT. VF, ventral furrow. (E, F) At later stages, *mir-9a* is expressed throughout the ectoderm. (G) WT *Drosophila* wing disc: *mir-9a* is expressed widely throughout the disc. (G′) Zoom of the area highlighted in (G). Scalebars: (A–G) 100 µm, (A′–D′) 10 µm, and (G′) 5 µm.

We have used a nascent transcript approach using smFISH to detect expression in cells that are actively producing the miRNA primary transcript (pri-miRNA). To do so we have designed sets of ∼48 probes (reported in supplementary material) that hybridize to a ∼1-kb region in the primary miRNA transcript flanking the *mir-9a* locus. This allows the detection of active *mir-9a* transcription in the cell nuclei as previously described by Aboobaker *et al.* (2005). We find that expression of *mir-9a* initiates in early stage 5, at which point it is expressed throughout the dorsal ectoderm of the embryo (Figure 1, A and A′). The pattern displays a precise boundary between actively transcribing cells and nonexpressing cells, which correlates with the mesoderm–ectoderm boundary similar to that described by Fu et al. (2014). There are also small domains at the posterior and anterior embryonic ends lacking *mir-9a* expression. Later, during *Drosophila* embryonic stages 6 and 7, *mir-9a* expression is maintained in this pattern throughout the ectoderm (Figure 1, B, B′, C, and C′), clearly marking the boundary between ectodermal cells and invaginating mesodermal cells. At stage 8, the mesoderm is almost completely invaginated and the *mir-9a* expression domain covers the embryo surface (Figure 1, D and D′), with the exclusion of the same regions at the anterior and posterior ends. At stages 11 and 14, *mir-9a* continues to be expressed throughout the ectoderm, except from a dorsal anterior region, and it is largely absent from the amnioserosa (Figure 1, E and F).

It has previously been reported that *mir-9a* is expressed widely in the third instar wing disc but not in cells expressing *sens* (Li *et al.* 2006; Biryukova *et al.* 2009). Similarly, we observed that *mir-9a* is actively transcribed throughout the wing disc (Figure 1G). We note that chromosomal pairing in imaginal tissue results in the two nascent transcription sites (TSs) merging into a single detectable spot (Figure 1G′).

### Immunodetection of Sens-sfGFP fusion protein allows the study of Sens expression in *Drosophila* embryos at single-cell resolution

**Figure 2:**
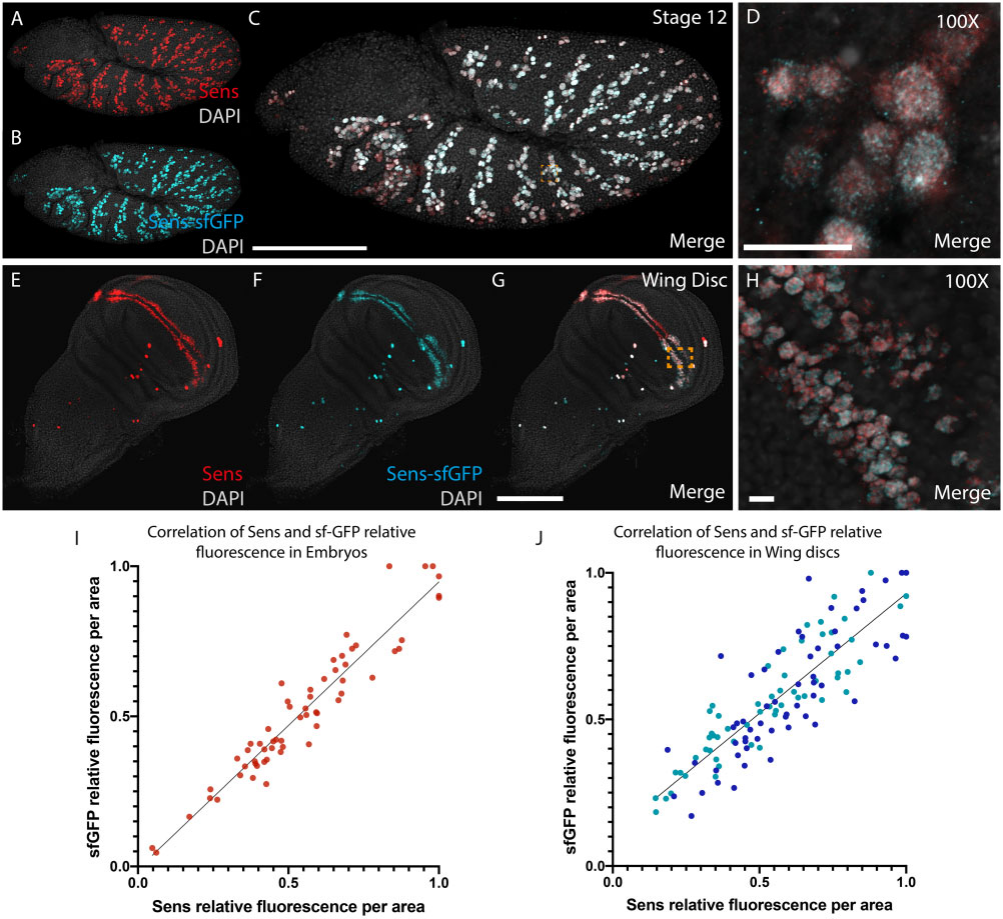
Co-localization of sfGFP reporter with endogenous Sens. Transgenic stage 12 embryo stained with antibodies against Sens (A, red) and sfGFP (B, cyan). The two antibodies co-localize (merged in D, E), showing that the sfGFP reporter is expressed in the same cells that are expressing endogenous Sens. Transgenic third instar larval wing disc stained with antibodies against Sens (E, red) and sfGFP (F, cyan). Again, the two antibodies co-localize in the same cells (merged in G, H). Correlation between relative fluorescence of Sens antibody and sfGFP antibody in embryos (I) and wing disc (J). For each of three embryos, fluorescence measurements were performed in 10 cells, while for each of three wing disc, 20 cells were measured. For each replicate, antibody fluorescence was normalized using the maximum value measured in that replicate. Scalebars: (C) 100 µm, (D) 10 µm, (G) 100 µm, and (H) 10 µm.

The role of *miR-9a* in regulating the transcription factor *sens* is well characterized genetically during SOP specification (Li *et al.* 2006; Cassidy *et al.* 2013). To investigate the dynamics of *miR-9a* regulation of *sens*, we developed a strategy to simultaneously observe *sens* transcription and protein accumulation at the single-cell level via confocal fluorescent microscopy. In principle, the efficient detection of proteins in fixed samples using IF relies on the availability of antibodies that can specifically detect the protein of interest. Alternatively, *Drosophila* lines that express the protein of interest fused to a reporter protein can be detected enzymatically or by fluorescence (Timmons *et al.* 1997; Chatterjee and Bohmann 2012). We have therefore used a transgenic *Drosophila* reporter line that, in addition to the endogenous *sens* locus, carries two additional copies of a C-terminally tagged Sens-sfGFP reporter that can be detected either directly (live imaging of GFP fluorescence) or by IF using either anti-GFP or anti-Sens antibodies (Sarov *et al.* 2016). We were therefore able to use two methods in order to examine Sens dynamics: direct detection of Sens using antibodies against the endogenous protein, and indirectly using anti-GFP antibodies. To validate that the reporter accurately reflects endogenous Sens protein pattern and level we performed a double staining experiment against endogenous Sens using an anti-Sens antibody (obtained from H. Bellen lab; Nolo *et al.* 2000), and an anti-sfGFP antibody, in both embryos and wing discs (Figure 2). Under these conditions, we expect that anti-Sens antibodies will detect protein products derived from all four *sens* loci (the two endogenous loci and the two sfGFP-tagged reporter loci inserted at the VK00033 landing site on the third chromosome), while anti-sfGFP antibodies will detect only proteins from the two sfGFP-tagged loci.

The co-localization of the WT and sfGFP-tagged signals during embryogenesis and wing disc development are shown in Figure 2. The data clearly show that during both embryogenesis (Figure 2, A–D) and wing disc development (Figure 2, E–H), the two signals co-localize in the same cells. In the wing disc, we also noticed a small number of cells with clear presence of Sens protein but reduced detection of sfGFP. This occurs particularly in cells near the intersection of the dorsal–ventral and anterior–posterior boundary. It is likely that detection of Sens is more robust in this dynamic region as Sens protein is produced from four loci. Given the minimal differences between Sens and sfGFP detection, we are confident that the reporter accurately reflects endogenous Sens expression patterns during embryonic and wing imaginal disc development. We further measured the relative fluorescence from each of the two antibodies in single cells to ensure that the reporter gene reflects Sens abundance. As expected, the sfGFP reporter is expressed proportionally to *sens* in both embryos (Figure 2D) and wing discs (Figure 2I). Taken together, these observations indicate that the fluorescence signal from antibodies against sfGFP provides reliable information on both endogenous Sens localization and relative expression levels.

### *mir-9a expression pattern* and *Senseless* protein levels are inversely correlated during embryogenesis

**Figure 3:**
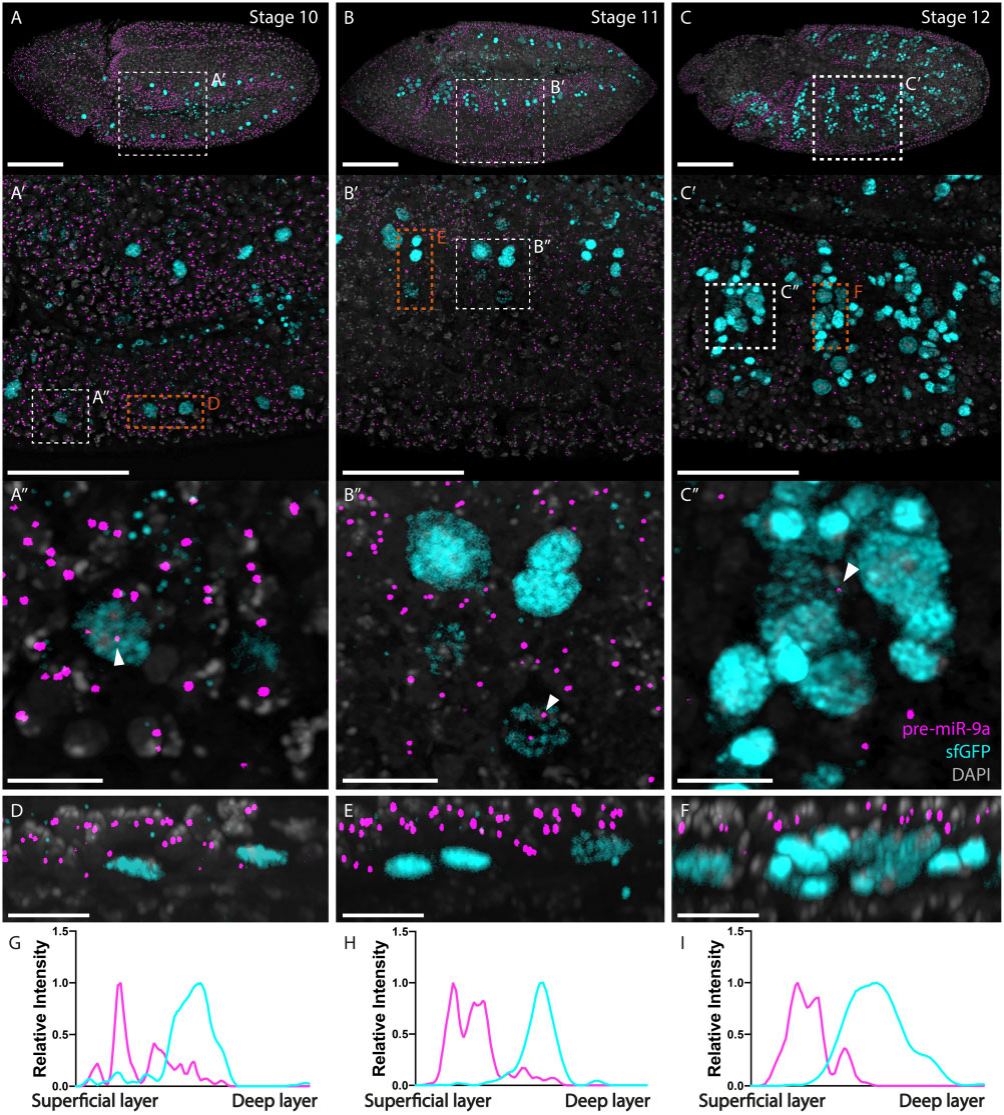
*mir-9a* is generally not actively transcribed in Sens-expressing cells during embryogenesis. (A–C) Transgenic embryos stained with probes against pri-*mir-9a* (magenta) and antibodies against sfGFP (cyan). (A) Stage 10 embryo. Sens is expressed in isolated cells. Some Sens-expressing cells have active sites of transcription for *mir-9a*, but these appear to be less intense. (A′) Zoom of highlighted area in (A). (A″) Zoom of highlighted area in (A′) (white line). (B) Stage 11 embryo. Sens is expressed in more cells, a few of which still transcribe *mir-9a*. (B′) Zoom of highlighted area in (B). (B″) Zoom of highlighted area in (B′) (white line). (C) Stage 12 embryo. Sens expression reaches its peak during embryogenesis and *mir-9a* is generally not transcribed in Sens-expressing cells. (C′) Zoom of highlighted area in (C). (C″) Zoom of highlighted area in (C′) (white line). (D–F) Orthogonal projections from highlighted areas in (A′–C′), respectively (orange lines). Superficial cellular layers at the top, deeper layers at the bottom. (G–I) Relative intensity of *mir-9a* TSs (magenta) and GFP (cyan) through the projections in (D–F), respectively. Data were normalized by dividing each value by the maximum value of the same channel. Scalebars: (A–C) 100 µm, (A′–C′) 50 µm, (A″–C″) 10 µm, and (D–F) 5 µm.

In order to study the reciprocal dynamics of *mir-9a* and *sens* during embryogenesis and wing disc development, we simultaneously tracked active sites of *mir-9a* transcription using smFISH and Sens abundance via IF. We investigated these patterns at three different stages of embryonic development: stages 10–12 (Figure 3, A–C) after early *sens* expression and the initial stages of SOP specification. Interestingly, we found that *mir-9a* transcription levels were inversely corelated with Sens protein levels, and that *mir-9a* transcription is repressed rapidly after the initiation of Sens expression. Intriguingly, we noticed that a very small number of Sens-expressing cells also displayed active *mir-9a* TSs (Figure 3, A″–C″). Moreover, when we look at the fluorescence levels of sfGFP and the size of *mir-9a* TSs in the subset of cells that express both, it is evident that both miRNA active transcription and Sens level are lower in comparison to the rest of the cells. We believe that *sens* has just started to be translated in these cells and *mir-9a* transcription is stopping. We also noticed that while cells transcribing *mir-9a* are mostly located on the most superficial cellular layers, Sens-expressing cells are located inwards, as reported in the orthogonal projections and fluorescence intensity measurements in Figure 3, D–F and G–I, respectively. Our interpretation of these results is that the accumulation of Sens in the nucleus is associated with repression of *mir-9a* transcription, either by direct repression, or through an intermediary negative regulator.

**Figure 4:**
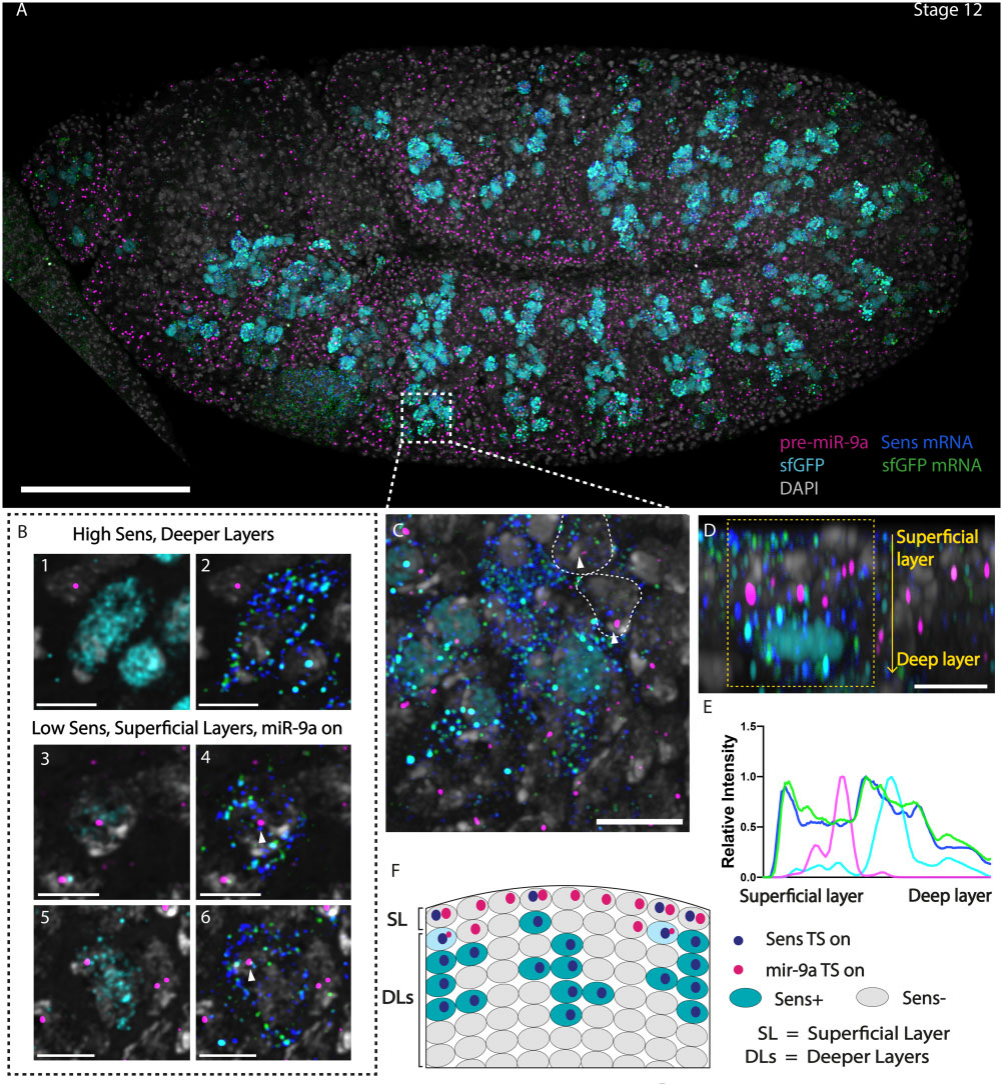
*miR-9a* is actively transcribed after *sens* transcription starts but before Sens protein is detectable. (A) Stage 12 transgenic embryo stained with probes against pri-*mir-9a*, sfGFP mRNA, and *sens* mRNA and antibody against sfGFP. (B) At deeper layers cells that are expressing Sens are not usually transcribing *mir-9a* (B1 and B2). At more superficial layers there are cells that are expressing Sens at low levels (B3 and B5) and that are transcribing *sens* and *mir-9a* (B4 and B6). (C) zoom from different focal plane showing that some cells transcribing *sens* are still transcribing *mir-9a* (highlighted with white arrows, white lines represent cell boundaries drawn from the DAPI channel). (D) Orthogonal view from (C) showing that *mir-9a* is mostly expressed in cells at the embryo surface, some of which show detectable *sens* and GFP mRNA. Cells that already have Sens protein have migrated inwards. (E) Relative intensity of *sens* mRNA (blue), GFP mRNA (green), mir-9a TSs (magenta), and GFP (cyan) across the area highlighted in panel (D). Data were normalized by dividing each value by the maximum value of the same channel. (F) Schematic representation of what is reported in panel (D). SL, superficial cellular layer, DLs, deeper cellular layers. Scalebars: (A) 100 µm, (B1–B6 and D) 5 µm, and (C) 10 µm.

To further investigate the dynamic relationship between expression of *mir-9a* and Sens, we developed a multichannel experiment to simultaneously study the expression pattern of *mir-9a* TSs and Sens-sfGFP with *sens* and *sfGFP* mRNAs (probes reported in supplementary material) (Figure 4, for panels A and C split channels are reported in Supplementary Figure S1 for detail). We observe that cells transcribing both *mir-9a* and *sens* have not yet accumulated significant quantities of Sens protein and are usually located in superficial layers, while cells with high Sens levels are usually not transcribing *mir-9a* and are located inwards (Figure 4, B and C). At this stage, Sens-expressing cells occupy several embryonic cellular layers ranging from the superficial to deeper layers. Using an orthogonal projection to clearly distinguish these embryonic cell layers, we can see that the cells containing Sens protein are located inwards, whereas cells that are transcribing both *mir-9a* and *sens* mRNA, but that have not accumulated Sens protein, are usually localized on the embryonic surface (Figure 4D). For a clearer visualization, we also plotted embryonic depth against average fluorescence intensity of the four channels (Figure 4E). We believe that the rapid dynamic changes in *mir-9a* expression are correlated with SOP differentiation, during which SOPs progressively migrate inwards as Sens protein accumulates and *mir-9a* is turned off.

### *mir-9a* is actively transcribed in cells containing Sens during early SO specification in the third instar imaginal wing disc

Since *sens* regulates SOP differentiation during PNS development in the *Drosophila* larvae (Singhania and Grueber 2014), we applied the approach outlined above to study *mir-9a* expression pattern and Sens abundance in third instar imaginal wing discs at the single-cell level. The adult *Drosophila* wing possesses a spatially organized series of bristles (a class of SO) located at the wing margin (Hartenstein and Posakony 1989). Flies in which Sens is ectopically expressed in the wing disc exhibit an increased bristle number in that wing region (Jafar-Nejad *et al.* 2003). During larval development, *sens* starts to be expressed at around 15 h after third instar ecdysis in single SOPs in the wing notum. At around 30 h, *sens* is expressed in an increased number of isolated SOPs in the wing and notum area plus in two distinct stripes of cells in the wing disc pouch (Mirth *et al.* 2009). Cells belonging to these two rows of Sens-expressing cells will give rise to the adult wing margin bristles (Jafar-Nejad *et al.* 2006).

**Figure 5:**
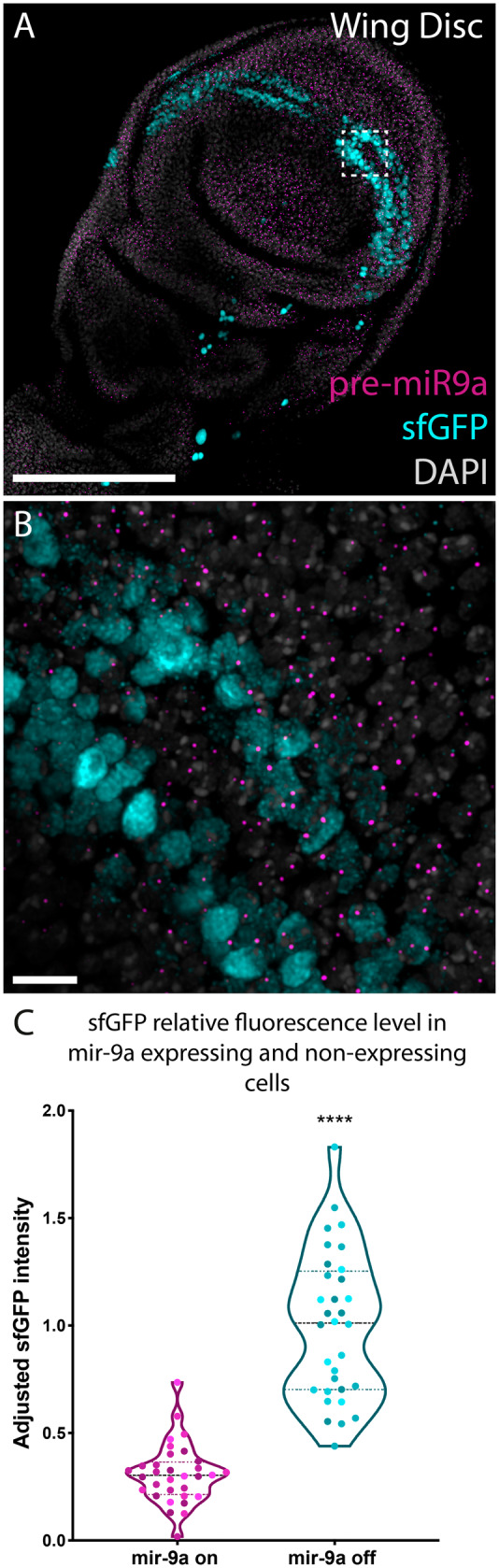
In the wing disc, Sens-expressing cells are generally actively transcribing *mir-9a*. (A, B) Third instar larval transgenic wing disc stained with probes against pri-*mir-9a* and antibody against sfGFP. Many cells that are actively transcribing *mir-9a* are also expressing Sens. (C) Adjusted sfGFP intensity coming from Sens-expressing cells that are and are not actively transcribing *mir-9a*. Different colors represent data coming from different disks (*n* = 4). Data from each replicate were normalized using the maximum adjusted fluorescence value from the group *mir-9a* off from that replicate. Scalebars: (A) 100 µm and (B) 10 µm.

By nascent transcript smFISH, we observed that *mir-9a* is expressed in nearly all cells in the wing disc. When we correlated *mir-9a* expression with that of Sens in third instar discs we identified a small population of Sens-expressing cells with no *mir-9a* expression. These cells always had high levels of Sens protein, similar to our observations in the embryo. We also observed that many cells that have low or intermediate Sens protein levels are actively transcribing *mir-9a* (Figure 5, A and B). It is interesting to note that *mir-9a* TSs size in Sens-expressing cells do not differ from the size of TSs belonging to cells that are not expressing Sens protein. This indicates that these cells may not shut down *mir-9a* transcription, which might be kept active or modulated via transcriptional bursting. Nonetheless, at this stage only a minority of cells that contain Sens protein are not transcribing *mir-9a*. We therefore measured the intensity coming from sfGFP antibody (a proxy for Sens protein levels) at the single-cell level to see if there was a difference in Sens levels between *mir-9a*-expressing and nonexpressing cells. The data clearly show that Sens is more abundant in cells that are not transcribing *mir-9a* (Figure 5C). However, our finding of concurrent expression of Sens and *mir-9a* contradicts aspects of the previously established model of triple row bristle specification (Li *et al.* 2006), which suggested a binary co-expression pattern.

## Discussion

The stable and reproducible development of the *Drosophila* PNS is an extraordinary model of how stereotyped stability of cellular differentiation is achieved (Jan and Jan 1994; Barad *et al.* 2011). In this study, we focused on the role of *Drosophila miR-9a* in regulating the function of *Senseless (sens)*, a crucial transcription factor that orchestrates SOP differentiation and PNS development in embryos and larvae. Dysregulated *miR-9a* expression results in disrupted *sens* function leading to altered SOP differentiation and loss of stable stereotyped neural development (Li *et al.* 2006; Cassidy *et al.* 2013). It has been hypothesized that Notch signaling plays a key role in regulating *mir-9a* transcription in epithelial cells adjacent to potential SOPs thus preventing accumulation of Sens and unintended differentiation of additional SOPs (Li *et al.* 2006). Despite extensive study of *sens* expression (Nolo *et al.* 2000; Mirth *et al.* 2009), there is little information regarding the developmental profile of *sens* and *mir-9a* co-expression.

**Figure 6:**
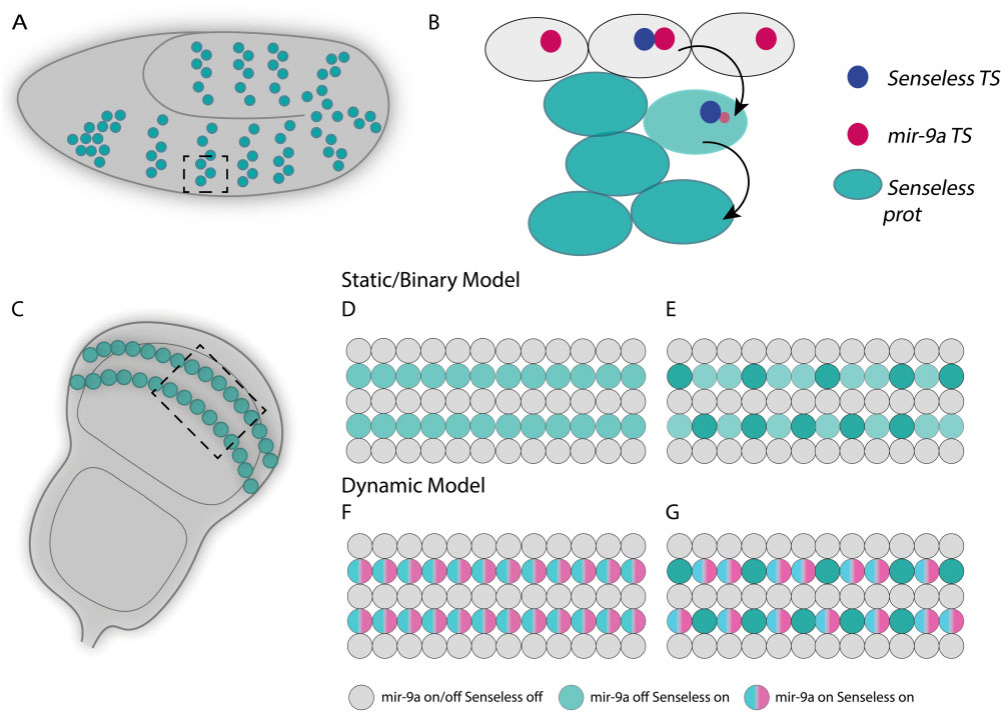
Model of *mir-9a* and *sens* interaction during embryogenesis and wing disc development. (A) In the embryo, *sens* is expressed in clusters of cells. (B) The orthogonal view of one of these clusters shows that *mir-9a* is transcribed in cells at the top, some of which are turning *sens* transcription on. As *sens* mRNA gets translated, these cells stop transcribing *mir-9a* and move inwards. (C) In the *Drosophila* wing disc, *sens* is expressed in two rows of cells (plus additional isolated cells, not shown). (D, E) In a static model, *mir-9a* and *sens* are not co-expressed. (F, G) Dynamic model in which all the cells that contain Sens are transcribing mir-9a, which is then turned off in a specific class of SOPs.

During embryonic development, we show that *mir-9a* is initially expressed throughout the neurogenic ectoderm, and a mutually exclusive expression pattern with *sens* is established. Our single-cell analysis shows that cells just initiating *sens* expression, who therefore have not accumulated measurable Sens protein, actively transcribe *mir-9a*. However once Sens protein levels increase, *mir-9a* transcription is lost. The data suggest that *mir-9a* expression is turned off when the level of Sens protein reaches a specific threshold. The intriguing possibility that the regulation may be direct cannot yet be confirmed. We do not observe any evidence that *miR-9a* expression is reinitiated during SOP cell lineage differentiation in the daughter cell and subsequently repressed as Sens levels rise. The co-expression dynamics are only observed in the selection of the initial SOP cell and, without subsequent repression by *miR-9a*, Sens protein accumulates rapidly (Figure 6, A and B). sfGFP staining also revealed the presence of Sens-expressing cells in different cell cycle stages (Figure 3C′), as previously reported for the differentiating R8 photoreceptor cells in the eye imaginal disc (Firth and Baker 2005; Ruggiero *et al.* 2012). We therefore suggest that *miR-9a* repression of Sens protein accumulation initially plays a regulatory role to buffer SOP specification and ultimately to stabilize the geometrical pattern of differentiating R8 cells. This negative feedback loop involving Sens and *miR-9a* may be key in the regulative establishment of the SOP pattern. It is currently unclear if *sens* directly switches off *mir-9a* transcription or if it is an indirect effect of a multilevel genetic cascade.

Li *et al.* presented evidence that in the third instar wing imaginal disc cells that express Sens do not express *mir-9a*, which is otherwise widely expressed throughout the disc (Li *et al.* 2006). We find that *mir-9a* and Sens are often though not always co-expressed in the wing disc. More specifically, we find that among the Sens-expressing cells, those that are also transcribing *mir-9a* present a lower level of nuclear Sens, similar to that seen fleetingly prior to the establishment of the terminal and mutually exclusive pattern of SOP specification in the early embryo. The main difference here is that this subset of cells in the wing disc does not seem to be stopping *mir-9a* expression as it was happening in the embryo. This suggests that *mir-9a* and *sens* have an intricate reciprocal dynamic expression during embryogenesis and larval development.

Our findings complement the model (Figure 6, D and E) presented by Li *et al.* (2006) and show that *mir-9a* and *sens* exhibit dynamic co-expression rather than a binary one. Our findings are also in concordance with the suggestion by Jafar-Nejad (2006) that the genes that orchestrate PNS development in embryos and larvae might be different, even though the process follows a similar pattern. For instance, during embryogenesis *achete* and *scute* are necessary for *sens* activation, while during larval development this function is accomplished by *Wingless* (Jafar-Nejad *et al.* 2006). *miR-9a* function might therefore differ between embryonic and larval SOP development via the presence or absence of other *miR-9a* targets. We propose that *mir-9a* repression during embryogenesis allows *sens* to reach a specific threshold in order to establish the correct number and pattern of embryonic SOPs. During larval development, *sens* might be expressed at different levels depending on the subclass of SO and this in part involves regulatory feedback by *miR-9a*. The fly wing margin possesses two different kinds of SO, the mechanoreceptors and chemoreceptors, and these have a very precise pattern (Hartenstein and Posakony 1989; Raad *et al.* 2016). *mir-9a* expression may be involved in a regulatory loop with *sens* to set different Sens levels and thereby control the kind of SO that will develop from *sens*-expressing cells that are not transcribing *mir-9a* and will adopt chemoreceptor SOP fate: chemoreceptors are lower in number than mechanoreceptors and their localization and alternation resembles the pattern of cells with high Sens expression level. Therefore, we believe that *miR-9a* serves to keep Sens expression low in mechanoreceptor precursor cells to ensure they adopt the correct SOP cell fate. Temporally, our model suggests that *mir-9a* is initially expressed in all *sens-*expressing cells, delaying the adoption of a specific SOP fate (Figure 6, F and G). As SOPs adopt their specific cell fate, *mir-9a* is switched off as a consequence of the transition from a multipotent precursor to a determined cell. Recent work on the evolution of sensory organ identity suggests that the gene network underlying specification is labile and complex (Klann et al. 2020). Future investigation to understand the possible role of *miR-9a* and Sens levels as upstream regulators of the expression of sensory organ identity genes may provide insight into this hypothesis.

Our work suggests that *mir-9a* has a dynamic role in the specification of SOP differentiation, tuning *sens* expression to ensure that the correct number of cells adopt the appropriate SOP fate. The molecular mechanism that dictates *mir-9a* transcription during PNS development remains unknown; its elucidation is important for complete understanding of this dynamic process. A fundamental question that needs to be answered is the mechanism by which the mutual regulation of *mir-9a* and *sens* act to establish the observed co-expression dynamic, switching from mutually exclusive to co-expressed depending on the fly developmental stage. This study demonstrates the importance of examining miRNA regulation and miRNA-target gene expression dynamics at a single-cell level in the developing organism. We suggest that these dynamic co-regulatory processes are a general feature of miRNA function during development.
