# Corrigendum to: Single-cell visualization of *mir-9a* and *Senseless* co-expression during *Drosophila melanogaster* embryonic and larval peripheral nervous system development

**DOI:** 10.1093/g3journal/jkab048

**Published:** 2021-04-07

**Authors:** Lorenzo Gallicchio, Sam Griffiths-Jones, Matthew Ronshaugen

**Affiliations:** 1 School of Biological Sciences, Faculty of Biology, Medicine and Health, The University of Manchester M13 9PT, UK; 2 School of Medical Sciences, Faculty of Biology, Medicine and Health, The University of Manchester M13 9PT, UK

In the article by L. Gallicchio, S. Griffiths-Jones, and M. Ronshaugen (G3, 11: 1; https://doi.org/10.1093/g3journal/jkaa010) entitled “Single-cell visualization of *mir-9a* and *Senseless* co-expression during *Drosophila melanogaster* embryonic and larval peripheral nervous system development”, corrections have been made to address the following errors:

All author affiliations have been updated from the following:

School of Biological Sciences, Faculty of Biology, Medicine and Health, The University of Manchester, Manchester Academic Health Science Centre, Manchester M139PT, UK

To the following, for First-author Lorenzo Gallicchio and Co-author Sam Griffiths-Jones:

School of Biological Sciences, Faculty of Biology, Medicine and Health, The University of Manchester M13 9PT, UK.

To the following, for Corresponding author Matthew Ronshaugen:

School of Medical Sciences, Faculty of Biology, Medicine and Health, The University of Manchester M13 9PT, UK.

Finally, the terms “Wing disk” and “disk” have been corrected throughout the manuscript to read “Wing disc” and “disc”.

